# Colposcopic and Histological Outcome of Atypical Squamous Cells of Undetermined Significance and Atypical Squamous Cell of Undetermined Significance Cannot Exclude High-Grade in Women Screened for Cervical Cancer

**DOI:** 10.31557/APJCP.2019.20.9.2579

**Published:** 2019

**Authors:** Osman Ortashi, Dana Abdalla

**Affiliations:** 1 *Sidra Medical and Research Center, Qatar, Specialist Physician, Womens Health Institute, Al Ain Hopital, United Arab Emirates.*

**Keywords:** Cervical smear, Pap smear, ASCUS

## Abstract

**Objectives:**

The objectives of the study are to assess the prevalence of colposcopic and histological abnormalities in patients diagnosed with ASCUS and ASC-H and to compare the prevalence of CIN in each group.

**Methods:**

Population-based cross-sectional retrospective study was conducted in one of tertiary hospitals in UAE. All cervical smears reported as ASCUS or ASC-H in 2015 were included in this study. The local guideline in 2015 was to refer all cases of ASC for colposcopy assessment.

**Results:**

Overall 7,418 cervical smears were processed at our laboratory service, 5.6% (n=413) were reported as ASC. 95% of them (n=394) were ASCUS and 5% (n=19) were ASC-H. The overall prevalence of high grade CIN in patients with ASC-H is 26% compared with 0.8% for patients with ASCUS regardless the age. The relative risk of patients with ASC-H is 8 folds higher than patients with ASCUS to have low grade CIN but 29 fold higher risk of having High grade CIN and the P value =0.0001.

**Conclusion:**

ASC-H cytology confers a substantially higher risk for high grade CIN than ASCUS regardless of age. HPV test is an important triage test in patients with ASCUS to predict cellular changes and CIN.

## Introduction

Organized cervical cancer screening has been proved to be one of the most successful cancer prevention strategies. Countries with established organized cervical cancer screening programs witnessed significant reduction in the incidence of cervical cancer of approximately 70%. (Luhn et al., 2007). Cervical smear or (Pap smear) smear is the standard screening test for cervical cancer and cervical dysplasia (Tambouret, 2013). The test was developed by Georgios Papanikolaou in 1941. The cervical smear test is based on collection of cells from the cervix and vagina and to detect cellular abnormalities that arise mainly from the transformation zone where almost all cervical dysplasia and cancers arise. The cervical smear which is screening test yields cytological result but not histological diagnosis.

The Bethesda System for reporting cervical cytology was developed in 1988, and was widely embraced after it was adopted by the National Cancer Institute in the same year. The system was revised and updated in 1991 and 2001 to provide a uniform system of terminology that would promote clear management guidelines (Davey 2003; Solomon, 2002). ASC referred to atypical squamous cells and this category includes both ASC-US (Atypical squamous cells of undetermined significance) and ASC-H (Atypical squamous cells, cannot exclude high grade squamous intraepithelial lesion). ASC represents 3.6% of all cervical smears (Gupta et al., 2007), ASCUS account for 2.8% of all cervical smears worldwide (Solomon et al., 2002), while ASC-H represents only 0.2% of cytological abnormalities as reported by the College of American Pathologists in 2003-2003 (Sherman et al., 2007).

ASC-H represents about 8.2% of all patients diagnosed with atypical squamous cells (ASC) which is approximately one in ten cervical cytological specimens that are interpreted as ASC (Srodon et al., 2006).

The objectives of the study are to assess the prevalence of colposcopic and histological abnormalities in patients diagnosed with ASCUS and ASC-H and to compare the prevalence of CIN in each group.

## Materials and Methods

This is population-based cross-sectional retrospective study was from Jan 2014 to Dec 2014 to look at colposcopic and histological abnormalities in patients diagnosed with ASCUS and ASC-H at one tertiary hospital in United Arab Emirates. The cytology department at this tertiary hospital provides the central service for the region. All cervical samples were collected by general practitioners and gynecologists using SurePath^®^ liquid-based cytology system (TriPath Imaging, Burlington, NC) and sent to the department of pathology within 2 days of collection. Upon arrival, all samples were assigned a microscopy number and then processed for cytological examination as per laboratory protocol. Certified cyto-technicians and cytopathologists make the diagnosis. Our inclusion criteria was all smears reported as ASCUS or ASC-H with completed charts and records while our exclusion criteria was ASCUS or ASC-H smears with missing or deficient charts and records. General consent was obtained from all patients at the time of cervical smear taken. 

**Table 1:** Histological Results of Patient with ASCUS and ASC-H Stratified by Age and HPV Testing

ASC	ASCUS n=394				ASC-H n=19		P value = 1.0
Age	<40 years n=178		≥40years n=216		<40 years n=8	≥40years n=11	
	HPV +	HPV -	HPV +	HPV -	3	2	
					15.8%	10.5%	
Low grade CIN	12	4	1	6			
	3%	1%	0.3%	1.5%			
High grade CIN	2	0	1	0	2	3	
	0.6%	0.0%	0.3%	0.0%	10.5%	15.8%	

**Table 2:** The Risk of CIN in Patients Diagnosed with ASCUS and ASC-H

		ASC		Relative Risk	95% CI	P-value
		ASCUS (n = 394)	ASC-H (n = 19)			
Histology	No abnormality	366	8	--Ref--	--	--
		93.1%	44.4%			
	CIN 1	24	5	8.06	2.82 to 23.07	0.0001
		6.1%	27.8%			
	CIN 2 and above	3	5	29.22		0.0001
		0.8%	26%		12.23 – 69.79	

A data sheet was designed to extract the following information: demographic data of the patients at the time of screening, colposcopic and histological results of patients with ASCUS and ASC-H, results of HPV infection in both groups and other risk factors like smoking and immune deficient status. All the data was obtained from the patient’s electronic file (CERNER) using the patient’s identification number (MRN) which is unique to every patient. CERNER is a medical records system that creates and maintains all patient data electronically. The system captures patient data, such as patient complaints, lab orders and results, medications, diagnoses, and procedures, as well as all demographic information of the patients. The cervical smear results were classified according to the Bethesda System. The ethical approval was obtained from the Human Research Ethics Committee at the college of medicine and health sciences at UAE University.

Descriptive statistics were used to analyze the demographic characteristics of all women diagnosed with ASCUS and ASC-H to compute the means and standard deviations (SD) for continuous variables such as age, parity and Body Mass Index (BMI). Prevalence of high grade CIN weather diagnosed by colposcopy or pathology was calculated for both groups (ASCUS and ASC-H). We stratified histological results of patient with ASCUS and ASC-H by age and HPV testing. Data analysis was performed using IBM SPSS Statistics version 21. 

## Results

Between Jan 2014 and Dec 2014 a total of 7418 cervical smears were processed at Tawam Hospital laboratory service, 5.6% (n=413) were ASC which include ASCUS and ACS-H; which represent our study sample. The mean age in our study group is 41±10.97 years and the mean of the parity is 4±3.04. Out of 413 patients diagnosed with ASC, 95% (n=394) were ASCUS and 5% (n=19) were ASC-H.

Table 1 shows that there is no statically significant difference in the rate of high grade CIN between women above and below the age of 40 in both ASCUS and ASC-H. No high grade CIN was seen in patients with negative HPV test in all groups however negative HPV test did not exclude low grade CIN. The prevalence of High grade CIN in patients with ASCUS and positive HPV test is 0.8% in all age groups compared with 0.0% in patients with ASCUS and negative HPV test. The overall prevalence of high grade CIN in patients with ASC-H is 26% compared with 0.8% for patients with ASCUS. 

Out of 413 patients with ASCUS and ASC-H there were 95.4% (n=394) patients with ASCUS. Colposcopy was performed in 34% (n=134) patients with ASCUS. Normal colposcopy was reported in 43% patients and only 1.5% of them had colposcopy features of high grade CIN. 4.6% (n=19) of screened cases were reported as ASC-H. Colposcopy was done in 73.7% (n=14) of them, normal colposcopy was found in 14.3% while 35.7% of them had colposcopy diagnosis of high grade CIN. 

Out of the 134 patients with ASCUS who had colposcopy; 41.8% (n=56) patients had cervical biopsy. High grade lesions were found in only 5.4%, on the other hand out of the 19 patients with ASC-H we found record of 14 patients who had colposcopy, 5 patients were missing. Out of the 14 patients who underwent colposcopy 92.9% (n=13) had cervical biopsy, high grade CIN was found in 38.5% of them. 

The relative risk of low grade CIN with ASC-H is 8 folds higher than patients with ASCUS while the relative risk of high grade CIN is 29 fold higher with ASC-H compare with ASCUS which was statistically significant as shown in Table 2. 

## Discussion

In this study the total cervical smears done during the study period were 7,418 smears. ASC represents 5.6% (n=413) from total cervical smears. 95% (n=394) were reported as ASCUS and 5% (n=19) were reported as ASC-H. This was not different from what was published in other studies. (Solomon et al., 2002; Apgar et al., 2003). In our sample we found ASC-H represented 0.26% of total studies cervical smears, Gupta and his team examined 29,475 cervical smears and they found that ASC-H representing 2.2%. (Gupta et al., 2007). In contrast nationwide survey by the College of American Pathologists conducted 2002-2003 found that ASC-H accounted for approximately 0.2% of cytological interpretation (Katki et al., 2013).

We found is no statically significant difference in the rate of high grade CIN between women above and below the age of 40 in both ASCUS and ASC-H which is similar to what was reported by by study carried out in Thailand by Kietpeerakool and his colleagues (Kietpeerakool et al., 2014), however some other studies showed younger patient age is a predictor of underling high grade CIN in women with ASC-H (Sung et al., 2011) which is also supported by findings of Selvaggi (2003) who stratified his study sample by age and found higher percentage of high grade CIN in 65% of premenopausal of women with ASC-H compared with 35% in the postmenopausal wome. The finding in our study of no difference in the prevalence of high grade CIN in women less and older than 40 years old with ASCH-H could be explained by small study sample

The overall prevalence of high grade CIN in patients with ASC-H is 26% compared with 0.8% for patients with ASCUS. The relative risk of low grade CIN in patient with ASC-H is 8 folds higher than patients with ASCUS. However patients with ASC-H has 29 fold higher risk of having high grade CIN compared with patients with ASCUS which is statistically significant. Most of the studies have confirmed that ASC-H confers significantly higher risk for high grade CIN than ASCUS. Kietpeerakoo found that the incidence of underling high grade CIN in an ASC-H smear 69.4% (Kietpeerakool et al, 2014) which is similar to what was found by Selvaggi 68% (Selvaggi, 2013). Duncan and Jacob (2005) found that the positive predictive value of ASC-H for histologically proven high grade CIN was 40.4%. Similar but higher percentage of of high grade CIN was reported by Mokhtar who found that 59.4% of the cases that were diagnosed as ASC-H found to have high grade CIN on subsequent biopsies. This correlation was stronger in patients below the age of 40 years (65.1% vs. 47.5%) (Mokhtar et al., 2008). However few studies reported lower chance of high grade CIN in patients with ASC-H like Nogara and colleagues who found that high grade CIN represented only 23% of total histology of the loop electrosurgical excision procedure (LEEP) done for patients with ASC-H (Nogara et al., 2011). These results conclude that ASC-H has significantly higher chance for worse histological outcome and needs an immediate colposcopic evaluation while ASC-US cases can be managed conservatively by repeating Pap smear as published in the guideline without a significant risk of missing a high grade lesion.

ASCUS represented 95.4% (n=394) patients. Colposcopy was performed in 34% (n=134) patients of them, normal colposcopic result was found in 43% patients and only 1.5% of these were having colposcopic features of high grade CIN. Cervical biopsy was done in 41.8% (n=56) patients. High grade lesions were found in only 5.4%of the patients who underwent biopsy. On the other hand ASC-H was found in 4.6% (n=19) patients. Colposcopy was done in 73.7% (n=14) of them, we could not trace the course of the other 5 who did not attend for colposcopy. Normal colposcopic result was found in 14.3% while 35.7% of them had high grade CIN. Cervical biopsy was done in 92.9% (n=13) patients, negative pathological result was found in 7.7% and high grade CIN was found in more than one third of them (38.5%). This almost the same as what Gupta found where 30.8% and 3.2% of their patients with ASC-H and ASCUS respectively had high grade CIN on biopsy, this results are almost similar to our study (Gupta et al., 2007). On the other hand, Rekhi found high grade CIN in 73.3% of 15 biopsies out of 20 cases diagnosed with ASC-H compared with 3.2% cases of high grade CIN in 23 biopsies out of 50 cases diagnosed with ASCUS, however this particular used conventional pap smears which explain why it results are higher than our study where Liquid-based cytology was used (Rekhi, 2010). 

Though the current strategies of screening for cervical cancer remain efficient, however these strategies have many limitations including lack of prognostic information, affordability and acceptability. The current trend now is look at certain biomarkers to improve the prognostic value as well as acceptability of cervical cancer screening. There is now emerging and encouraging date on the use of *Importin-β*, *exportin-5*, *p16*, *Ki-67*, *Mcl1*, *PDL1*, and *cFLIP* as biomarker for CIN 1 lesion as they have been reported to show over-expressed in most of these lesions (Nicol et al., 2019; Huang et al., 2012). 

The strength of this study is that it looked at under studied category of abnormal cervical smears, also the study relied on an accurate electronic records, on the other hand the study was limited by the small size. 

In conclusion, ASC-H cytology confers a substantially higher risk for high grade CIN than ASCUS regardless of age. HPV test is an important triage test in patients with ASCUS to predict cellular changes and CIN.
